# Early Differentiated CD138^high^MHCII^+^IgG^+^ Plasma Cells Express CXCR3 and Localize into Inflamed Kidneys of Lupus Mice

**DOI:** 10.1371/journal.pone.0058140

**Published:** 2013-03-08

**Authors:** Stéphanie Lacotte, Marion Decossas, Carole Le Coz, Susana Brun, Sylviane Muller, Hélène Dumortier

**Affiliations:** CNRS, Institut de Biologie Moléculaire et Cellulaire, Immunopathologie et Chimie Thérapeutique/Laboratory of Excellence Medalis, Strasbourg, France; Institut National de la Santé et de la Recherche Médicale U 872, France

## Abstract

Humoral responses are central to the development of chronic autoimmune diseases such as systemic lupus erythematosus. Indeed, autoantibody deposition is responsible for tissue damage, the kidneys being one of the main target organs. As the source of pathogenic antibodies, plasma cells are therefore critical players in this harmful scenario, both at systemic and local levels. The aim of the present study was to analyze plasma cells in NZB/W lupus mice and to get a better understanding of the mechanisms underlying their involvement in the renal inflammation process. Using various techniques (i.e. flow cytometry, quantitative PCR, ELISpot), we identified and extensively characterized three plasma cell intermediates, according to their B220/CD138/MHCII expression levels. Each of these cell subsets displays specific proliferation and antibody secretion capacities. Moreover, we evidenced that the inflammation-related CXCR3 chemokine receptor is uniquely expressed by CD138^high^MHCII^+^ plasma cells, which encompass both short- and long-lived cells and mostly produce IgG (auto)antibodies. Expression of CXCR3 allows efficient chemotactic responsiveness of these cells to cognate chemokines, which production is up-regulated in the kidneys of diseased NZB/W mice. Finally, using fluorescence and electron microscopy, we demonstrated the presence of CD138^+^CXCR3^+^IgG^+^ cells in inflammatory areas in the kidneys, where they are very likely involved in the injury process. Thus, early differentiated CD138^high^MHCII^+^ rather than terminally differentiated CD138^high^MHCII^low^ plasma cells may be involved in the renal inflammatory injury in lupus, due to CXCR3 expression and IgG secretion.

## Introduction

Systemic lupus erythematosus (SLE) is the antibody-mediated autoimmune disease *par excellence*. Indeed production of autoantibodies (autoAb), among which anti-nuclear autoAb (ANA), is a hallmark of lupus. Depending on their antigenic specificity, autoAb can be of diagnostic value (e.g. anti-Sm and anti-DNA Ab), but some of them are also closely correlated to disease activity and have been incriminated for their key contribution to the autoimmune pathogenesis, such as autoAb specific for double-stranded DNA or nucleosome, which are involved in the development of lupus nephritis [1], [2].

When talking about autoAb, one logically thinks of autoreactive B lymphocytes and their terminal differentiation stage, namely plasma cells. This differentiation process is obviously central to the pathology of autoimmune diseases such as lupus. It has long been thought that short-lived plasma cells were continuously newly generated during autoimmune responses and therefore were responsible for autoAb production [3]. However, not only short-lived but also long-lived plasma cells have been demonstrated to significantly contribute to chronic humoral autoimmunity in lupus mice [4]. The involvement of long-lived plasma cells is also supported by the observation that circulating (auto)Ab levels mostly remain unaffected despite classical aggressive immunosuppressive treatments or, more recently, despite administration of CD20-targeting Ab (Rituximab), which depletes mature and memory B cells but not long-lived plasma cells [5], [6].

Classical view regarding humoral immune responses holds that long-lived plasma cells are generated as the result of a differentiation process initiated in lymphoid organs upon secondary antigen(Ag)-specific encounter of B cells with helper T cells within germinal centers. These cognate interactions give rise to proliferating precursors of plasma cells, namely plasmablasts that will terminally differentiate into Ab-secreting non-dividing plasma cells, the main final destination of which is the bone marrow [7], [8]. There, they can survive for very long periods of time, thanks to specific niches that provide them with an appropriate complex molecular microenvironment [9]. Within this environment, CXCL12, which is secreted by bone marrow stromal cells, is of particular importance since it participates to the recruitment of plasma cells. Indeed it has been demonstrated that upon their differentiation process into plasma cells, B lymphocytes change their chemokine receptor expression profile, which allows them to emigrate from the spleen and to travel towards different tissues [10], [11]. In particular, expression of CXCR4 is required for the recruitment of Ab-secreting cells in the bone marrow, whereas CCR9 and CCR10 allow IgA-producing cells to specifically home into mucosal tissues [12]. CXCR3, the receptor for the inflammation-related chemokines CXCL9, CXCL10 and CXCL11, has also been shown to be expressed on memory B cells and plasma cells during secondary immune responses both in human and mice [13], [14].

Although the overall picture of plasma cell differentiation seemed to be clearly established, some studies have underscored an unexpected complexity of plasma cells regarding their survival, homing and consequently their functional properties [15], [16]. This heterogeneity of the plasma cell compartment is likely to exist in autoimmune settings. However, relatively few data are available regarding plasma cell behavior in autoimmune diseases such as SLE. Increased numbers of plasmablasts and plasma cell precursors have been measured in the blood of children and adults with SLE [17], [18]. Some years ago, Jacobi *et al*. [19] have described a correlation between HLA-DR^high^CD27^high^ plasmablasts and disease activity in SLE patients. In the (NZBxNZW)F1 (NZB/W) mouse model of lupus, plasma cells, including cells secreting pathogenic autoAb, have been evidenced not only in lymphoid organs but also in inflamed kidneys [20]–[22]. Interestingly, high numbers of long-lived plasma cells remain in the spleen of these animals [4].

In this context, the initial aim of the present work was to get more deeply into the plasma cell differentiation process in lupus. To address this question, we thoroughly characterized the phenotype and function of plasma cell-related subsets, which are localized in the spleen of diseased NZB/W mice. This led us to identify a non-terminally differentiated CD138^high^MHCII^+^ plasma cell population, which might be central in the development of lupus nephritis. Indeed, the majority of these cells secrete IgG and express CXCR3, which may directs them towards inflamed kidneys where the cognate chemokines are produced.

## Results

### Identification of Four Phenotypically Distinct B Cell/Plasma Cell Subsets in the Spleen of Diseased Lupus Mice

**Figure 1:**
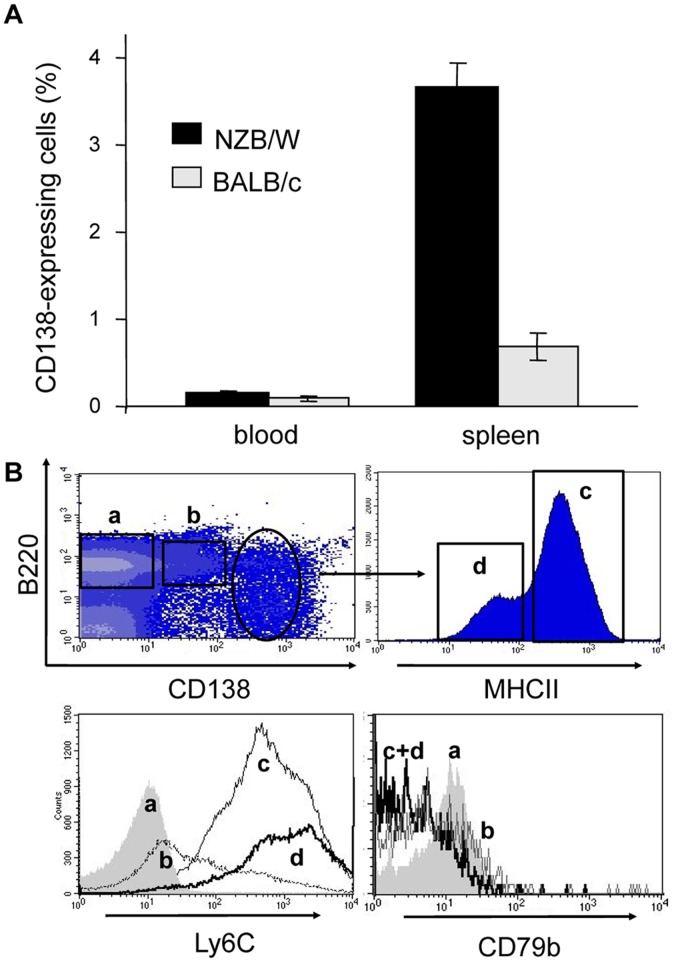
B cell/plasma cell subsets in diseased lupus NZB/W mice. A) Percentage of CD138-expressing cells among living cells in the blood and spleen of 32–36 week-old NZB/W mice and BALB/c mice (10 and 4 mice per group respectively) as determined by flow cytometry analysis. Results are expressed as mean % +/− SEM. B) Representative FACS figures showing four main populations of cells in the spleen of a sick NZB/W mouse according to surface expression of key B cell and plasma cell markers: (a) B220^+^CD138**^−^**Ly6C**^−^**CD79b^+^ cells, (b) B220^+^CD138^int^Ly6C^int^CD79b^int^ cells, (c) CD138^high^MHCII^+^Ly6C^+^CD79b**^−^** cells and (d) CD138^high^MHCII^low^Ly6C^+^CD79b**^−^** cells. This phenotypic analysis was performed in eleven 32–36 week-old proteinuria-positive NZB/W mice with similar results.

Previous work had described the presence of large numbers of Ig-secreting cells in the spleen of lupus mice [23]. We first analyzed the percentage of plasma cells, identified according to their expression of CD138 (syndecan-1), in the spleen and in the blood of 32–36 week-old proteinuria-positive lupus NZB/W mice and control age-matched BALB/c mice. As shown in Figure 1A, CD138^+^ cells were hardly detectable in the blood of both NZB/W and BALB/c mice. In contrast, we observed that in the spleen of lupus mice almost 4% of cells express CD138 as compared to less than 1% in the spleen of healthy BALB/c mice.

From these first results, we then performed a more thorough flow cytometry analysis of the cell populations present in the spleen of diseased NZB/W mice, according to the expression of classical markers for B lymphocytes and plasma cells, namely B220 and CD138, respectively. As shown for a representative mouse in Figure 1B, three cell populations could be identified based on the expression of these two markers, namely i) B220^+^ cells, which are likely to be regular B lymphocytes (a; 45%), ii) B220^+^ cells that express intermediary levels of CD138 (B220^+^CD138^int^; b; 7%) and iii) cells that express high levels of CD138 (4%). Knowing that downregulation of MHCII on the cell surface is associated with a terminal plasma cell differentiation stage [24], we further analyzed MHCII expression by CD138^high^ cells and we found that two subsets could be further defined within these cell populations (Figure 1B), namely CD138^high^MHCII^+^ (c; 3% of total spleen cells) and CD138^high^MHCII^low^ (d; 1% of total spleen cells). These results suggest a differentiation pathway from B220^+^ B lymphocytes to an intermediary double positive B220^+^CD138^int^ stage to CD138^high^ plasma cells, first expressing and then downregulating, MHCII molecules. This hypothesis was reinforced by the increasing expression of the Ly6C molecule through “a” to “d” cell subsets (Figure 1B, geometric mean fluorescence intensities measured in one representative experiment: a/3; b/50; c/464; d/834). Indeed, Ly6C was described as being a marker of plasma cells in mice [25]. The B cell antigen receptor, however, seems to be downregulated on CD138^high^ cells as shown by the absence of CD79b (Igβ) at the surface of the “c” and “d” subsets (Figure 1B).

**Figure 2:**
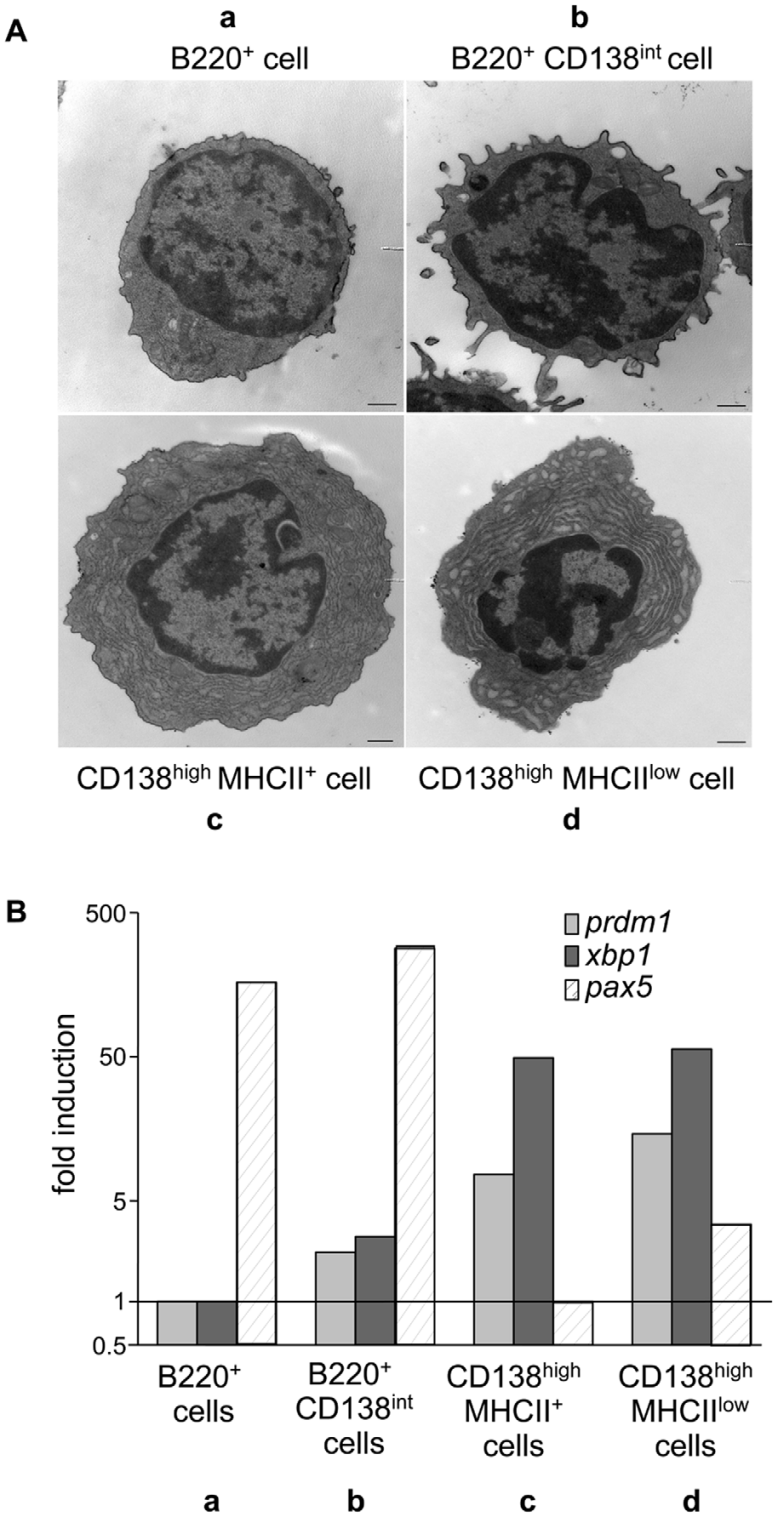
Characteristic ultrastructural morphology and transcription factor expression in cell subsets identified according to B220/CD138/MHCII expression. A) The four spleen cell subsets (a to d, as described in Figure 1) were sorted by flow cytometry from the spleen of diseased NZB/W mice. Cells were then embedded for electron microscopy observation. Images show one representative cell (out of 20 observed) in each panel. A representative experiment out of two is shown. Scale bars = 500 nm. B) Total RNA was isolated from each FACS-sorted cell subset and the expression of *prdm1* (Blimp-1; grey bars), *xbp1* (dark grey bars) and *pax5* (hatched bars) transcripts was evaluated by quantitative real-time PCR. Results are expressed as the fold induction of gene transcription as compared to the cell subset expressing the lowest transcript amount (B220^+^ cells as for *prdm1* and *xbp1*, and CD138^high^MHCII^+^ cells as for *pax5)*. All differences between raised values in each subset are statistically significant except for *pax5* when comparing the two B220^+^ subsets and the two CD138^high^ subsets to each other, respectively. Data of one representative experiment out of three are shown.

Next to the characterization of their surface phenotype, we studied by electron microscopy the ultrastructural morphology of these four cell subsets. For that purpose, we first sorted them by FACS based on the expression of B220, CD138 and MHCII. Figure 2A shows a representative image of one cell of each of the four cell subsets. The so-called “a” subset-derived cell harbors a large nucleus but few cytoplasm whereas the “c” and “d” cells have a larger diameter and an expanded cytoplasm filled with rough endoplasmic reticulum. These morphological characteristics correspond to those classically described for B lymphocytes and plasma cells, respectively. The double positive B220^+^CD138^int^ cell (“b” subset) looks similar to the B220^+^ cell, except that it displays more cytoplasmic prolongations.

In order to ascribe a B cell or plasma cell signature to our cell subsets, we performed a real-time PCR analysis of genes encoding three key transcription factors, which are known to control the differentiation pathway from B cell to plasma cell, namely *prdm1* (Blimp-1 protein), *xbp1* and *pax5*. Blimp-1 has been described as necessary and sufficient to drive final plasma cell differentiation, and is responsible for *pax5* repression and *xbp1* induction [26]. Data presented in Figure 2B show that B220^+^ cells, expressing CD138 or not (“a” and “b” subsets), express the highest levels of the mRNA encoding Pax5, which reveals their B-cell identity. On the contrary, high levels of Blimp-1 and Xbp1 mRNAs but low levels of Pax5-encoding mRNA are detected in CD138^high^ cells (“c” and “d” subsets), which rather correlates with a plasma cell phenotype.

Altogether, our data support the existence of four phenotypically distinct cell subsets in the spleen of NZB/W mice with installed disease, ranging from classical B cells (B220^+^ cells) to terminally-differentiated plasma cells (CD138^high^MHCII^low^ cells) through intermediary stages defined as B220^+^CD138^int^ and CD138^high^MHCII^+^ phenotypes.

### Each Cell Phenotype Possesses its Individual Functional Profile

**Figure 3:**
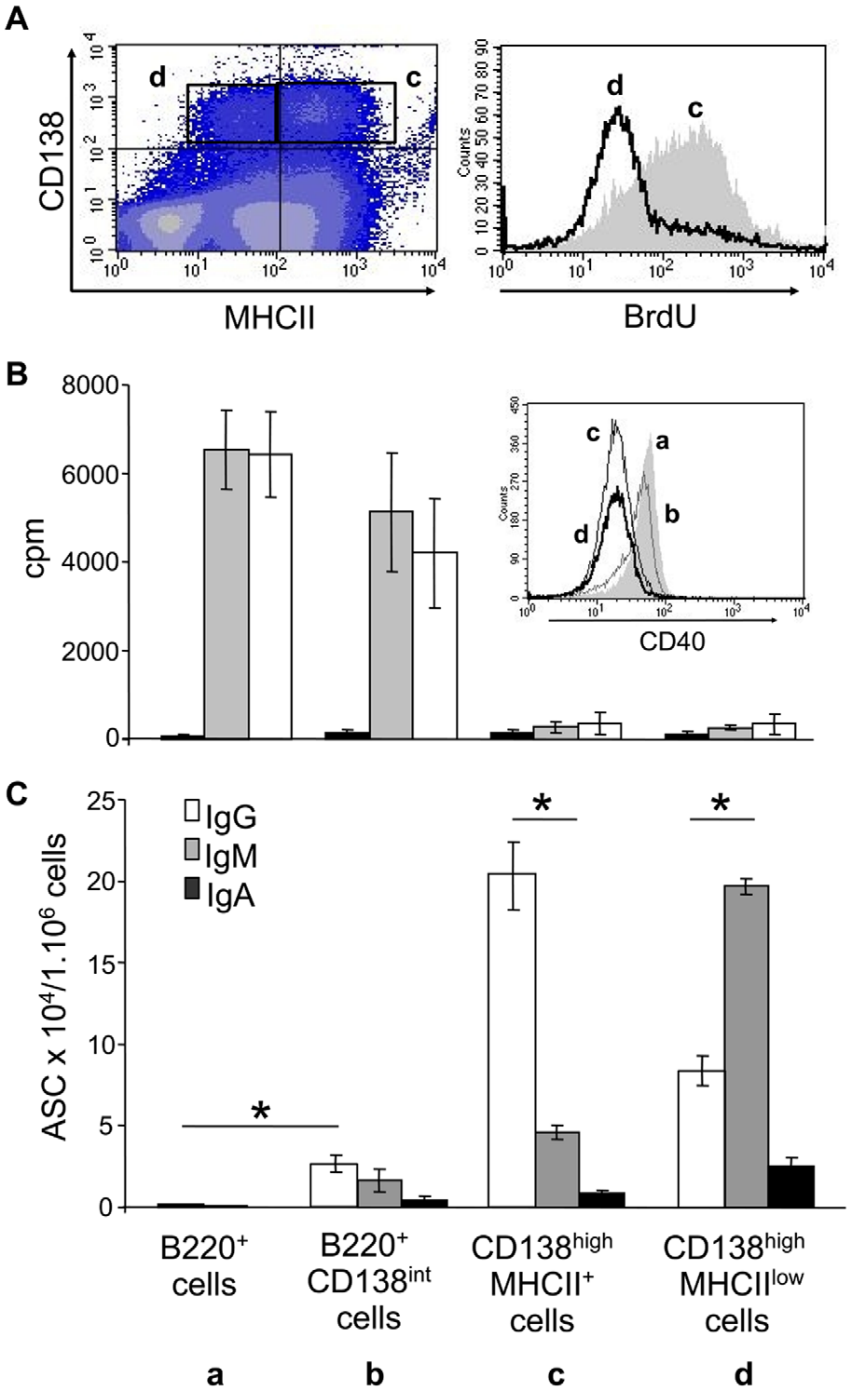
Each cell subset displays distinct proliferative and secretory abilities. A) *In vivo* proliferation upon BrdU administration to diseased NZB/W mice. Upon intra-peritoneal injection and five days feeding with BrdU, mice were killed and spleen cell subsets were analyzed for proliferation by flow cytometry. Among CD138^high^ cells, mostly MHCII^low^ cells (“d” subset) did not incorporate BrdU as shown on the histogram. B) and C) The four spleen cell subsets (a to d, as described in Figure 1) were sorted by flow cytometry from the spleen of diseased NZB/W mice. (B) Sorted cells were incubated for 3 days without stimulation (black bars), with LPS (gray bars) or with agonistic anti-CD40 Ab (white bars). Proliferation was measured upon tritiated thymidine uptake for 18 h and results are expressed as mean cpm +/− SEM. In the insert is shown the expression of CD40 by the four spleen cell subsets. (C) Cells were tested for spontaneous Ig secretion by ELISpot upon a 6 h-culture period. The three main Ig isotypes were tested: IgG (white bars), IgM (grey bars) and IgA (black bars). Results are expressed as the number of Ab-secreting cells (ASC) per 1×10^6^ cells (mean +/− SEM). *statistically significant differences of interest (*p*<0.05, Mann-Whitney U test).

Once we had phenotypically identified these cell subsets in the spleen of diseased NZB/W mice, we analyzed their functional properties. We focused our study on two main criteria, which differ from B lymphocytes to terminally-differentiated plasma cells, namely proliferation and Ig secretion. We first analyzed the *in vivo* spontaneous proliferation of the cells upon short-time BrdU administration to NZB/W mice (Figure 3A). Upon five days of exposure, we noticed that a majority of CD138^high^MHCII^low^ cells (“d” subset) had not incorporated BrdU, suggesting that they had not proliferated during this time period, which fits with the non-proliferative capacities of differentiated plasma cells. In contrast, a high proportion of CD138^high^ MHCII-expressing cells had incorporated BrdU within the five day period, suggesting either that they had themselves proliferated or that they were derived from proliferating cells. Other B-cell subsets (B220^+^ MHCII^+^) also incorporated BrdU (data not shown).

We then studied the *in vitro* proliferative response of each of the four subsets in response to classical B cell stimuli, namely lipopolysaccharides (LPS) and agonistic anti-CD40 Ab FGK-45. Each subset was sorted from diseased NZB/W mice by FACS and cultured with these stimulatory molecules for 3 days. As depicted in Figure 3B, no spontaneous proliferation was detected in these experimental conditions for any of the four cell subsets. Both LPS and anti-CD40 Ab induced a strong proliferation of the B220-expressing populations (i.e. “a” and “b” subsets) but not of the CD138^high^ cells (i.e. “c” and “d” subsets). One possible explanation resided in the fact that CD138^high^ cells may not express the appropriate receptors (TLR4 and CD40, respectively). We addressed this question and indeed we found that CD40 is present at low levels at the surface of these cells, which explains their non-responsiveness to FGK-45 Ab (see insert in Figure 3B). In summary, the “a” and “b” subsets behave like B lymphocytes whereas the “c” and “d” subsets are unable to proliferate in response to B cell stimuli and as such, behave like plasma cells. If we combine our *in vivo* and *in vitro* results, we may therefore conclude at this stage that the CD138^high^ MHCII-expressing cells (“c” subset) stem from the proliferating B220^+^CD138^int^ cell population and are not able to respond to T-cell derived signals anymore.

The major characteristic of plasma cells is their capacity to secrete Ig. In order to determine the potential secretory ability of each of the four cell subsets, we performed an ELISpot assay with cells sorted by flow cytometry beforehand. No stimulus was added and IgG, IgM and IgA isotypes were tested, as increased numbers of cells capable of secreting these three isotype autoAb are found in the peripheral blood of patients with SLE [27]. As expected, B220^+^ cells (“a” subset, regular B lymphocytes) did not secrete Ig at all (Figure 3C). Interestingly, the B220^+^CD138^int^ cell population (“b”) contained a small number of Ig-secreting cells. Therefore, this subset keeps its proliferative capacity while acquiring some secreting properties, which supports our hypothesis that they correspond to an intermediate differentiation stage. Finally, the two CD138^high^ cell subsets were unambiguously those with the highest Ig secretion capacity, which again supports their plasma cell status. However, very interestingly, we observed that most CD138^high^MHCII^+^ cells present in the spleen of lupus mice secrete IgG while a majority of CD138^high^MHCII^low^ cells are IgM-producers (confirmed by ELISA, not shown). This result was unexpected as we rather anticipated that the presumably final CD138^high^MHCII^low^ plasma cell differentiation stage would mainly consist in class-switched IgG-secreting cells, especially in autoimmune settings. On the contrary, we found that large numbers of spleen-resident plasma cells apparently do not go through the class switch process and may not all issue from the early differentiated CD138^high^MHCII^+^ subset, although it cannot be excluded that IgG^+^CD138^high^MHCII^low^ cells have migrated out of the spleen.

### CXCR3 is Preferentially Expressed by CD138^high^MHCII^+^ IgG-secreting Plasma Cells

**Figure 4:**
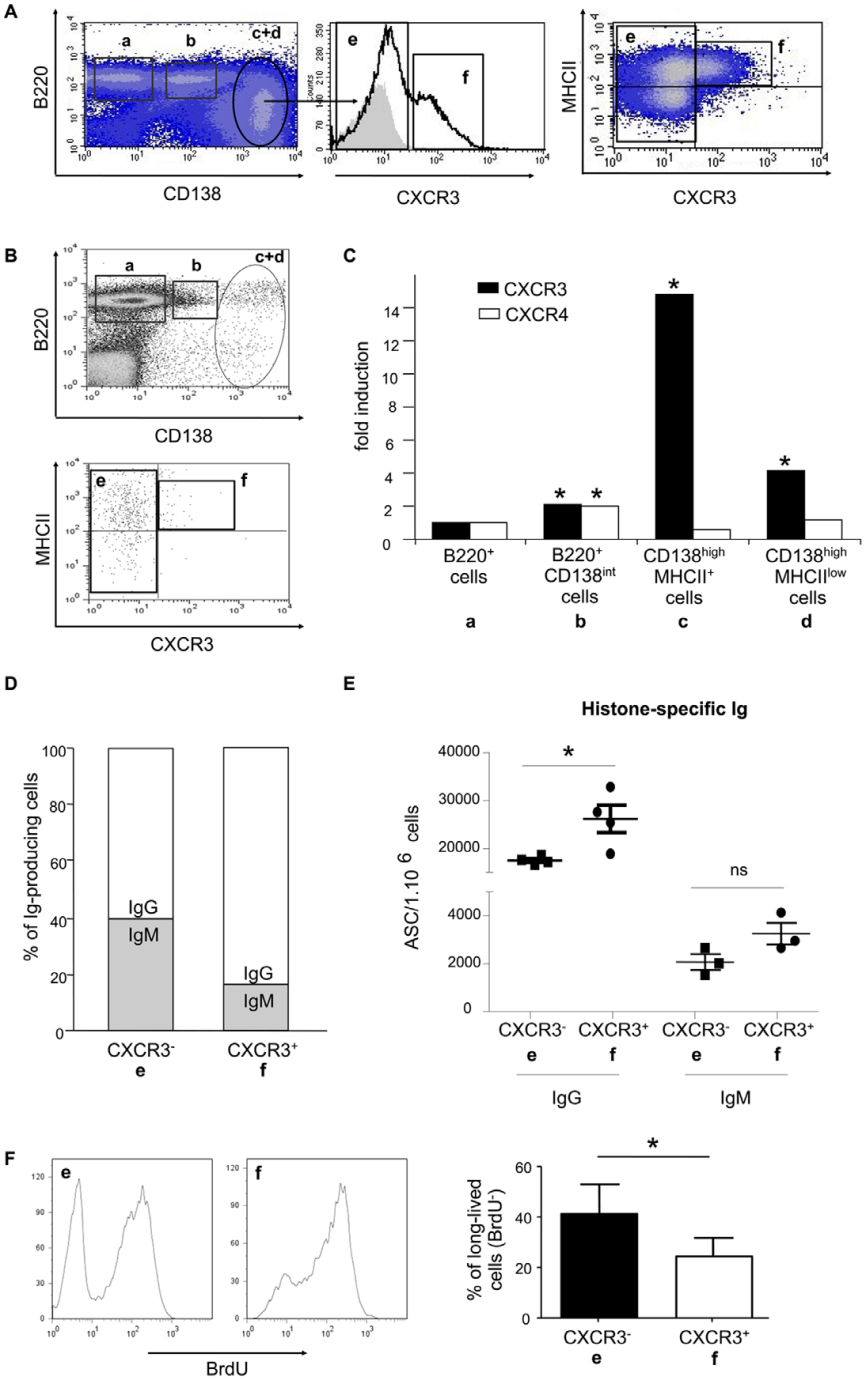
CD138^high^MHCII^+^ cells preferentially express CXCR3, produce IgG autoantibodies and encompass some long-lived cells. A) CD138-enriched spleen cells of diseased NZB/W mice were analyzed for CXCR3 expression by flow cytometry. Cells were co-stained with anti-B220, anti-CD138, anti-MHCII and anti-CXCR3 Ab. Among CD138^high^ cells, two subsets can be defined: one which does not express CXCR3 (e) and one which does (f) (see FACS histogram; grey area corresponds to the staining obtained with an isotype control Ab). All CXCR3^+^ cells belong to the MHCII^+^ subset of CD138^high^ cells (dot-plot, right). FACS data are derived from one representative NZB/W mouse out of eleven tested 32–36 week-old mice. B) Spleen-derived plasma cells (CD138^high^, c+d, upper dot-plot) from 30 week-old control BALB/c mice were analyzed for MHCII and CXCR3 expression (lower dot-plot). FACS data are derived from one representative BALB/c mouse out of 3. C) The four spleen cell subsets (a to d, as described in Figure 1) were sorted by flow cytometry from the spleen of diseased NZB/W mice. Total RNA was isolated from each subset and the expression levels of *cxcr3* (black bars) and *cxcr4* (white bars) transcripts were evaluated by quantitative real-time PCR. Results are expressed as the fold induction of gene transcription as compared to the “a” subset (B220^+^ cells). D) The profile of Ig isotypes produced by CXCR3**^−^** and CXCR3^+^ CD138^high^ cells, respectively, was assessed by specific IgM and IgG intracellular stainings. Surface stainings were performed with anti-CD138 and anti-CXCR3 Ab. Mean percentages of IgM- and IgG-producing cells among CD138^high^CXCR3**^−^** and CD138^high^CXCR3^+^ cell subsets are shown. Results are derived from eight individual NZB/W mice. E) CXCR3**^−^** (e) and CXCR3^+^ (f) cell subsets were sorted by flow cytometry from the spleen of diseased NZB/W mice and they were tested for spontaneous secretion of anti-histone IgG by ELISpot. Results are expressed as the number of ASC per 1×10^6^ cells (mean +/− SEM). Each dot corresponds to a different number of plated cells. Results are derived from three representative diseased NZB/W mice. F) Identification of short-lived and long-lived plasma cells within the CXCR3**^−^** (e) and CXCR3^+^ (f) subsets. Upon intra-peritoneal injection and two weeks feeding with BrdU, 36–38 week-old NZB/W mice were killed and spleen cell subsets were analyzed for BrdU incorporation by flow cytometry. FACS histograms are representative of one NZB/W mouse out of five. Percentages of long-lived (BrdU**^−^**) cells are expressed as the mean +/− SD (n = 5). *statistically significant differences (*p*<0.05, Mann-Whitney U test).

The CXCR3 chemokine receptor is known to mediate the recruitment of T cells to sites of inflammation and it has been suggested that it may also play a role in the localization of plasma cells generated during memory immune responses [13], [28]. Based on these data, we analyzed the presence of the CXCR3 receptor on the cell subsets identified in the spleen of sick NZB/W mice. As shown in Figure 4A, about 25% of CD138^high^ cells expressed CXCR3 (« f » subset). More importantly, it was striking to note that the vast majority of CXCR3^+^ cells belonged to the CD138^high^MHCII^+^ cell population and not to the CD138^high^MHCII^low^ subset. The “a” and “b” B220^+^ cell subsets did not express detectable levels of CXCR3 and the few CD138^high^ cells detected in the spleen of healthy BALB/c mice expressed CXCR3 to a much lesser extent (<10% of the “c+d” cells, Figure 4B). These results were confirmed and extended with a real-time PCR analysis using mRNA isolated from each of the sorted cell subset (“a” to “d”; Figure 4C). The highest levels of CXCR3-encoding transcripts were indeed found in CD138^high^MHCII^+^ cells (“c” subset; fold induction >14).

We also examined the relative expression levels of CXCR4, since CXCR4 and its ligand, CXCL12, are considered to be deeply involved in the migration of differentiated plasma cells toward bone marrow [10]. Surprisingly, the levels of CXCR4-encoding transcripts did not show major variations among the four cell subsets, the B220^+^CD138^int^ cells being the only ones for which the calculated fold of induction reached a value of 2. Of note, we could not detect the CXCR4 receptor at the surface of any of these subsets by flow cytometry (data not shown).

Having demonstrated on the one hand that CD138^high^MHCII^+^ cells were mainly IgG producers, and on the other hand that they were the only CD138^high^ cells expressing CXCR3, we then wanted to confirm the link between IgG production and CXCR3 expression. For that purpose, we performed intracellular IgM and IgG stainings to determine which isotype was preferentially produced by CD138^high^CXCR3^+^ and CD138^high^CXCR3**^−^** cells respectively. This allowed us to show that more than 80% of CXCR3-expressing plasma cells produce IgG (Figure 4D), which may confer them with a potential pathogenic role at the inflammation site. Indeed, IgG are usually considered as the most harmful isotype in autoimmune settings (especially IgG2 isotypes in NZB/W mice; [29]). Moreover, autoAb can be detected among these IgG, as shown for anti-histone autoAb in Figure 4E. Similar results were obtained for anti-chromatin autoAb (data not shown).

Finally, we investigated whether the “e” (CD138^high^CXCR3**^−^**) and “f” (CD138^high^CXCR3^+^) subsets we had identified, belonged rather to the short-lived or the long-lived classically described plasma cells. We thus administered BrdU to diseased NZB/W mice for 2 weeks, in order to differentiate proliferating BrdU^+^ short-lived plasma cells from the non-proliferating BrdU**^−^** long-lived ones (Figure 4F). Interestingly, some long-lived cells were detected among the CD138^high^CXCR3**^−^** subset (40% +/− SD of the “e” subset were BrdU**^−^** after 2 weeks). Even more importantly, 25% of the CXCR3-expressing cells harbored a long-lived phenotype, which means that MHCII expression does not directly correlate to the longevity status of plasma cells, and that not only short-lived but also long-lived cells can be recruited to inflammatory sites.

### CD138^high^MHCII^+^Cells Migrate Towards CXCR3-binding Chemokines, which are Up-regulated in the Kidneys of Diseased NZB/W Mice

**Figure 5:**
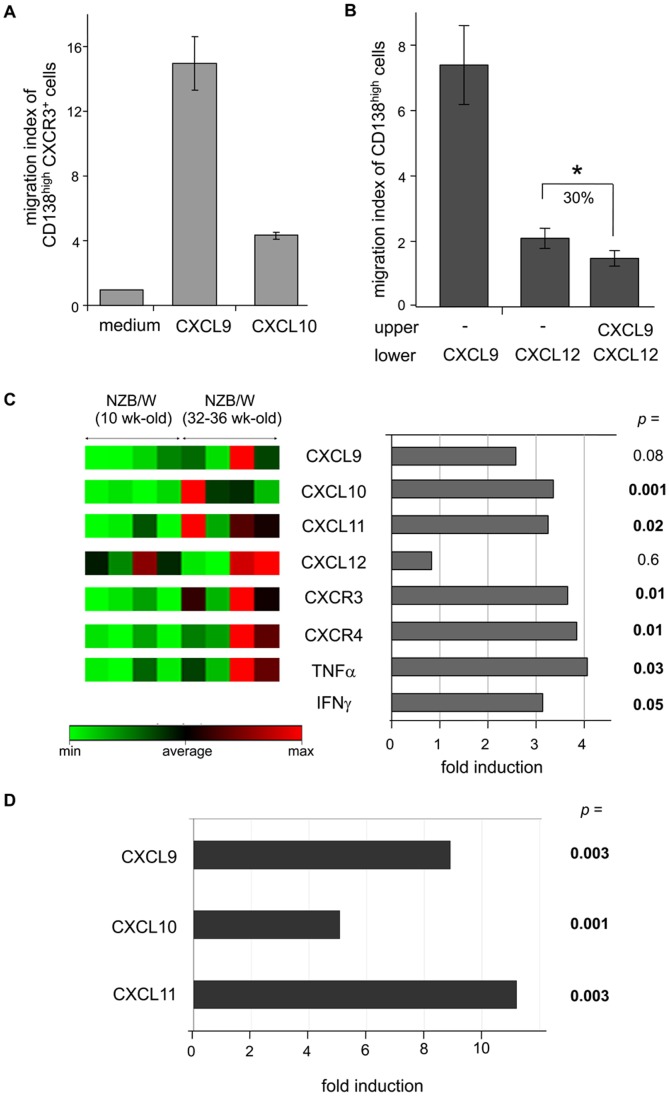
Migration of CD138^high^CXCR3^+^ plasma cells towards inflammation-related chemokines, which are overexpressed in the kidneys of diseased NZB/W mice. A) CD138-enriched splenocytes of NZB/W mice were tested for their chemotactic responsiveness to CXCL9 and CXCL10 in a Transwell migration assay. After a 2 h-incubation, cells which had migrated (lower chamber) were harvested and analyzed by flow cytometry (CD138/CXCR3 co-staining). Results are expressed as a migration index calculated as follows: % migrated CXCR3^+^CD138^high^ cells in the presence of chemokine divided by % migrated CXCR3^+^CD138^high^ cells in the control well (culture medium without chemokine). Data shown correspond to the mean migration index value derived from three independent experiments (each experiment being performed with a pool of cells isolated from two mice). B) CD138-enriched splenocytes of NZB/W mice were tested for their chemotactic responsiveness to CXCL9 and CXCL12 (lower chamber) in the presence or absence of a competitor chemokine (upper chamber). After 2 h of incubation, cells which had migrated (lower chamber) were harvested and analyzed by flow cytometry (CD138 staining). Results are expressed as a migration index and the percentage of inhibition of CD138^high^ cell migration is shown. *p<0.05 (Student t test). C) Total RNA was isolated from kidneys of 10 week-old (n = 4) and 32–36 week-old (n = 4; proteinuria-positive) NZB/W mice, and the expression of some chemokine, cytokine and chemokine receptor transcripts was evaluated by quantitative real-time PCR using the “Mouse Chemokines & Receptors” RT^2^ Profiler™ PCR Array (except for IFNγ transcripts). Only the results, which were directly related to the present work, are shown. *(Left panel)* Expression statistics for each gene were mapped to a color scale. Green color corresponds to low expression, whereas red corresponds to high expression of the transcripts. *(Right panel)* Results are expressed as the fold induction of gene transcription in 32–36 week-old diseased NZB/W mice as compared to 10 week-old NZB/W mice (Mann-Whitney U test). D) Real-time quantitative PCR analysis of transcripts encoding CXCL9, CXCL10 and CXCL11 in the kidneys of 35–40 week-old diseased NZB/W mice (n = 3; proteinuria-positive) and old, age-matched, healthy BALB/c mice (n = 3). Results are expressed as the fold induction of gene transcription in NZB/W mice as compared to control BALB/c mice (Mann-Whitney U test).

Since we had detected the presence of CXCR3 specifically at the surface of CD138^high^MHCII^+^ cells, we then checked whether this receptor was functional and could mediate the migration of these cells towards its cognate chemokines, namely CXCL9, CXCL10 and CXCL11. Results raised by Transwell experiments are presented in Figure 5A and show that many more CD138^high^CXCR3^+^ cells have migrated towards CXCL9 and CXCL10 (15 and 4 times more, respectively) than towards medium alone, highlighting their responsiveness to these pro-inflammatory chemokines and consequently their potential capacity to migrate to inflammatory sites. No migration was observed towards CXCL11 (data not shown). Of note, similar data were obtained when looking at CD138^high^MHCII^+^ instead of CD138^high^CXCR3^+^ cells, showing that the potential chemokine-induced CXCR3 internalization did not affect the results [30]. We also studied the capacity of CD138^high^ cells (“c” and “d” subsets) to respond to CXCL12 (Figure 5B) and found that, especially when compared to CXCL9, they harbor a weak sensitivity to this chemokine (migration index = 2), which correlates with the low CXCR4 expression we described above. Interestingly, in those experimental settings, the majority of migrating CD138^high^ cells, both towards CXCL9 and CXCL12, belong to the CD138^high^MHCII^+^ cell subset (not shown). This observation does not only confirm the presence of functional CXCR3 at their surface but also suggests that, although weak, the expression of the CXCR4 receptor by these cells is sufficient to mediate their migration. We then analyzed whether CXCL9 and CXCL12 could compete with each other in inducing CD138^high^ cell migration. Figure 5B shows that the migration signal given by the CXCL9 chemokine can somehow overcome the CXCL12 signal, which might mimic what happens *in vivo* when inflammation occurs.

As the inflammation-related CXCR3 receptor is functionally expressed by a subset of CD138^high^ cells and since kidneys are one of the main organs where inflammatory phenomena occur in lupus mice and patients, we then decided to analyze by quantitative real-time PCR the expression levels of genes encoding CXCR3-binding chemokines in the kidneys of sick NZB/W mice (32–40 week-old and proteinuria-positive) as compared to kidneys of young NZB/W animals (10 week-old, proteinuria-negative) or to kidneys of age-matched control BALB/c mice (35–40 week-old). We found a clear up-regulation of the three main CXCR3 ligands, namely CXCL9, CXCL10 and CXCL11 (fold change between 2 and 10) in the kidneys of diseased NZB/W mice compared to either kidneys of young non-diseased animals (Figure 5C) or to kidneys of old control BALB/c mice (Figure 5D), indicating that this up-regulation is directly related to disease development. Production of these chemokines is known to be induced by IFNγ and accordingly, we detected some up-regulation of this pro-inflammatory cytokine as well as of TNFα (Figure 5C, p<0.05). We also found a statistically significant increase of CXCR3 itself (p = 0.01), which is likely due to the presence of CXCR3-expressing cells in the kidneys of diseased mice. In contrast, no change in CXCL12 expression could be detected, although CXCR4 transcripts get expressed in a higher proportion when the disease develops (fold change = 3.8).

### CXCR3-expressing Plasma Cells Localize into Inflamed Kidneys

**Figure 6:**
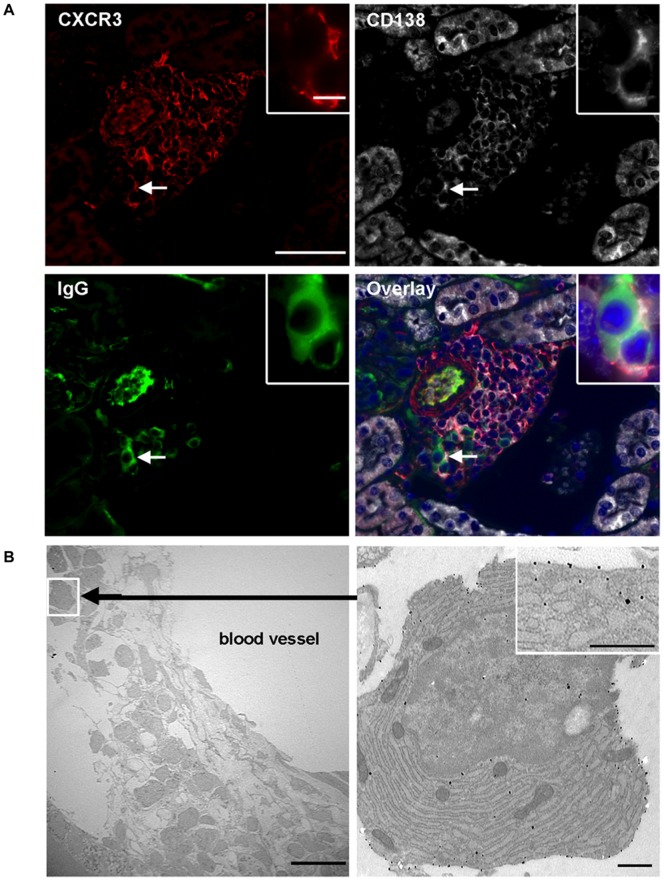
CXCR3^+^CD138^+^IgG^+^plasma cells are located in inflamed kidneys. A) IgG-producing CXCR3-expressing CD138^+^ cells were detected by immunofluorescence on paraffin-embedded kidney sections from proteinuria-positive NZB/W mice. Sections were labelled with CXCR3-specific (red), CD138-specific (white) and IgG-specific (green) Ab. Triple staining as well as nucleus DAPI staining (blue) are shown in the overlay picture. Scale bars: 50 µm (low magnification) or 10 µm (enlarged images). B) Cells showing the ultrastructural characteristics of plasma cells were visualized by transmission electron microscopy in the kidneys of nephritic NZB/W mice. The right-side image shows that these endoplasmic reticulum-rich cells express CXCR3 (revealed by gold-labeled anti-CXCR3 Ab). Scale bars: 10 µm (left image) and 500 nm (right images). All images are representative and derived from kidneys of two to four NZB/W mice.

Although our results seemed to indicate that CXCR3^+^CD138^high^ cells may be able to home into inflamed kidneys of NZB/W mice where CXCR3-binding chemokines are produced, the direct evidence of the presence of these cells in the kidneys of lupus mice was still missing. For that purpose, we performed triple immunofluorescence stainings on kidney slices from proteinuria-positive NZB/W mice. It allowed us to undoubtedly underscore the existence of CD138^+^ cells, which express CXCR3 and stain positive for cytoplasmic IgG, in the inflammatory perivascular infiltrates (Figure 6A). As expected [31], these cells are surrounded by large numbers of CXCR3-expressing T cells (see Figure S1). The final evidence was brought by analyzing nephritic NZB/W kidneys by electron microscopy (Figure 6B). Images clearly show the presence of infiltrated endoplasmic reticulum-rich plasma cells, which express the CXCR3 molecule at their surface as indicated by the presence of gold beads. Altogether our data strongly argue for the involvement of CXCR3-expressing IgG-producing plasma cells in the development of lupus renal disease.

## Discussion

The existence of a functional and maturational heterogeneity of the plasma cell pool has been well demonstrated in regular immunization-related circumstances, but little is known regarding what happens in autoimmune settings. Therefore, the initial goal of this study was to delineate the phenotype and function of plasma cell-related subsets, which are localized in the spleen of lupus-prone NZB/W mice. Based on the CD138 marker expression, we were able to identify three subsets, one expressing intermediary levels of CD138 (B220^+^CD138^int^) and one expressing high levels of CD138, the latter being further divided into CD138^high^MHCII^+^ and CD138^high^MHCII^low^ cells. Considering its proliferative and secretory abilities, the B220^+^CD138^int^ subset is extremely similar to what is usually called early plasmablasts. CD138^high^ cells, however, correspond to more differentiated plasma cells, as confirmed by their expression pattern of transcription factors and their high Ig-producing capacities. CD138^high^MHCII^low^ cells display a phenotype of terminally matured plasma cells, which correlates with the observation that they mostly are non-dividing cells, as shown by the low BrdU short-term incorporation *in vivo* and their lack of proliferation to polyclonal stimuli *in vitro*. Finally, the CD138^high^MHCII^+^ subset, which represents more than half of the CD138^high^ population, is likely to be an intermediate stage between proliferating plasmablasts and terminally differentiated plasma cells. They are potent Ig producers but still express MHCII molecules as a sign of immaturity. Moreover, we have shown that they lack BCR and CD40 expression, which makes them unable to respond to antigen encounter and costimulatory T cell help. However, they are labeled upon a 5-day BrdU feeding *in vivo*, suggesting that they rapidly arise from the previously described expanding plasmablast population.

Regarding this latter subset of non-terminally differentiated CD138^high^MHCII^+^ plasma cells, we found that they show very specific characteristics directly affecting their homing properties, and consequently their potential involvement in lupus pathology. Indeed, they express CXCR3, which is known to be central to inflammatory responses [32]. Within the four B cell/plasma cell-related subsets we identified in the spleen of diseased NZB/W mice, CD138^high^MHCII^+^ cells are the only ones displaying high CXCR3 levels. Interestingly, this CXCR3^+^ subset contains both short- and long-lived cells, as shown in a 2 week *in vivo* BrdU incorporation experiment, which strongly suggests that long-lived cells are involved in inflammatory lesions. Our results are in accordance with those of Hoyer *et al*. [4] and Starke *et al*. [22] who respectively showed that high numbers of long-lived plasma cells are present in the spleen and in the kidneys of NZB/W mice.

CXCR3 and its ligands have been widely implicated in the recruitment of T cells in target organs in varied inflammatory autoimmune disorders [33]–[35] and immune-mediated kidney diseases among which lupus glomerulonephritis [31], [36]. Steinmetz *et al*. [31] have indeed shown that, in MRL lpr/lpr mice made deficient for CXCR3, the trafficking of effector Th1 and Th17 cells to injured target organs is impaired, leading to reduced kidney tissue damage. However, B lymphocytes and plasma cells were not analyzed in their study, which means that the observed disease improvement may not be exclusively attributed to an impact on T cells but also on B cells and their derivatives. Up to now, only few data have been published regarding CXCR3 expression by cells of the B cell lineage in autoimmune settings. Up-regulation of CXCR3 was described on a substantial fraction of peripheral blood B cells from rheumatoid arthritis patients [37], [38] and more importantly, recruitment and accumulation of CXCR3-expressing plasma cells was described in the synovium of patients at early stages of this disease [39]. Similarly, Marques *et al*. [40] recently demonstrated that CXCR3 mediates Ab-secreting cell accumulation in the central nervous system during viral encephalomyelitis. Altogether, it seems that CXCR3 confers plasma cells with the capacity to migrate to inflamed tissues where they are likely involved in pathogenic mechanisms through their primary function i.e. the production of (auto)Ab. Our present work strongly suggests that the same mechanism is involved in the development of Ab-mediated nephritis in lupus. The antigen specificity of CXCR3-expressing Ab-secreting cells, which are detected in inflamed kidneys, remains to be fully characterized, but we have shown here that there are significantly more anti-histone secreting cells within the CXCR3^+^ than within the CXCR3**^−^**CD138^high^ subset in the spleen. We have also recently evidenced that kidneys of diseased NZB/W mice contain cells, which secrete autoAb recognizing the N-terminal region of histone H2B, a major lupus autoantigen [21]. It is therefore very tempting to hypothesize that these cells belong to the CXCR3-expressing MHCII^+^CD138^high^ subset presently described. Interestingly, autoreactive cells specific for the Sm autoantigen have been shown to be enriched in a subset of circulating CD19^high^ plasma cell precursors expressing high levels of CXCR3, in lupus patients with poor clinical outcome [41]. These cells are likely to home to sites of inflammation where they may play a pivotal role in disease pathogenesis, although contrary to anti-nucleosome autoAb, anti-Sm autoAb have not yet been proven to be pathogenic. With regard to autoAb pathogenicity, it is interesting to note that the majority of CXCR3-expressing plasma cells we identified in the spleen of NZB/W mice have undergone class-switch recombination and produce IgG. Such IgG^+^CXCR3^+^ cells are also found in inflamed kidneys as shown in this study by co-localization experiments. There, they are likely to participate to renal pathology by locally producing IgG autoAb known to be potentially responsible for tissue lesions, either via direct deposition or through ligation of immune complexes by FcγR [42], [43]. Such a preferential co-expression of CXCR3 and IgG has already been reported [44] and may be attributed to a cascade induction during class switch recombination, but also to environmental factors. Indeed the molecular link between CXCR3 and IgG might be the pro-inflammatory Th1 cytokine IFNγ, known to induce expression of both CXCR3 and its ligands as well as to favor class switch recombination towards IgG2a and IgG3 isotypes in mice, although rather surprisingly IgG1 was found to be the preferential CXCR3-associated isotype in human memory B cells [14]. In line with this result, CXCR3-deficient NZB/W mice were found to exhibit reduced production of total and anti-dsDNA Ab of the IgG1 subclass [45]. However, very surprisingly, CXCR3 defect led neither to reduced kidney cellular infiltrates nor to diminished glomerulonephritis in those animals, although, as we show in the present study, all elements (receptor/ligand/pathology-involved cells) are gathered to support a pathogenic role of this inflammation-related axis in the lupus-associated renal disease. It cannot be excluded that some compensatory mechanisms occurred in CXCR3-knockout mice, as already evidenced in other gene-deficient animals [46], [47]. CXCR4 and its ligand CXCL12 may take over as suggested by the beneficial effect of a CXCR4 antagonist peptide in another mouse model of lupus (B6.*Sle1Yaa*; [48]). However, we could not highlight any clear difference in CXCR4-encoding mRNA expression levels in the B/plasma cell populations we identified. Moreover, CXCL12 seems to be expressed in NZB/W kidneys but independently of disease severity, in accordance with what was suggested by Katrin *et al* [46]. Hopefully, injection of CXCR3 antagonists [49] to NZB/W mice should help clarifying this issue (underway in our laboratory).

Finally, another potential mechanism underlying the presence of Ab-secreting cells in inflamed kidneys may consist in their local generation in ectopic germinal centers. Such functional lymphoid structures have been described in several autoimmune conditions [50]–[52] among which the kidneys of a chemically-induced mouse model of lupus [53], and they could be the site where CXCR3-expressing early-differentiated plasma cells originate from. These cells might then be retained in the kidneys due to the local production of IFNγ-induced inflammatory chemokines CXCL9, CXCL10 and/or CXCL11. In conclusion, the prevalence or concomitant existence of these mechanisms (local generation *versus* migration of CXCR3^+^ early-differentiated plasma cells) as well as their pathogenic relevance in lupus, clearly deserve very interesting additional investigations.

## Materials and Methods

### Ethics Statements

Animal experiments were conducted in accordance with the European Community guidelines (Directive 2010/63/UE) on the protection of animals used for scientific purposes. The IBMC animal house facilities are approved by French veterinary services (#E67-482-2). All experimental protocols were carried out with the approval of the local Institutional Animal Care and Use Committee (CREMEAS).

### Mice and *in vivo* BrdU Experiments

Female BALB/c and NZB/W mice were purchased from Harlan (Gannat, France) and maintained in our animal facilities. In some experiments, NZB/W mice were injected i.p. with 200 µg of 5-bromo 2-deoxyuridine (BrdU, Sigma-Aldrich, St. Louis, MO) and then fed with BrdU (0.8–1 mg/mL in drinking water) for 5 days (short-term proliferation experiments) or fifteen days (identification of long-lived plasma cells).

### Antibodies and Flow Cytometry

Fluorescein isothiocyanate (FITC)-labeled anti-IA^d^ (AMS-32.1) and anti-CD40 (3/23), phycoerythrin (PE)-labeled anti-IA^d^ (AMS-32.1) and anti-CD138 (281-2), and anti-hamster IgG (G70-204+G94-90.5), peridinin chlorophyll protein (PerCP) Cy5.5-labeled anti-B220 (RA3-6B2) and streptavidin, biotinylated anti-Ly6C (AL-21) and anti-CD138 (281-2), and unlabeled anti-CD79b (HM79b) Ab were purchased from BD Pharmingen (San Diego, CA). Allophycocyanin (APC)-labeled anti-CXCR3 (220803) was purchased from R&D System (Abingdon, UK). FITC-labeled polyclonal goat anti-mouse IgG and anti-mouse IgM were purchased from Southern Biotechnology Associates (SBA, Birmingham, UK). Both intracellular Ig and BrdU stainings were performed using commercial kits from BD Pharmingen, according to recommended instructions (Cytofix/Cytoperm and BrdU Flow Kit, respectively). Cells were analyzed using a FACSCalibur™ apparatus and the CELLQuest™ software (BD Biosciences).

### Cell Preparation and Sorting

Single cell suspensions were prepared from spleen and bone marrow using 100 µm-cell strainers (Falcon, BD Biosciences, San Jose, CA). Red blood cells were lysed using a hypotonic solution. When necessary, CD138^+^ cells were enriched from total spleen cells by magnetic cell sorting. Briefly, cells were stained with anti-CD138 Ab for 15 min at 4°C and further incubated with magnetic microbeads coupled to anti-PE Ab, as indicated by the manufacturer (Miltenyi Biotec, Bergisch, Gladbach, Germany). CD138^+^-labeled cells were then selected upon cell loading on a MACS LS column (Miltenyi Biotec). In most experiments, the enriched fraction was collected and stained with anti-B220 and anti-IA^d^ Ab. Sub-populations of interest were then further sorted to more than 95% purity using a FACSDiva™ cytometer (BD Biosciences).

### Cell Culture and *in vitro* Polyclonal Activation

Sorted cell populations were incubated (1×10^5^ cells per well) with either anti-CD40 (FGK-45) Ab (5 µg/mL) or LPS (10 µg/mL; from *Escherichia coli* strain 0111.B4; Sigma-Aldrich) or were left untreated as a control. Cultures were performed in RPMI 1640 (Lonza, Verviers, Belgium) supplemented with 10% (v/v) fetal calf serum (Dutscher, Brumath, France), 10 µg/mL gentamicin (Lonza), 10 mM HEPES (Lonza) and 0.05 mM β-mercaptoethanol (complete medium). Cell proliferation was measured upon 72 h with [^3^H]-thymidine (1 µCi/well; specific activity 6.7Ci/mmol; PerkinElmer, Boston, MA). Thymidine incorporation was assessed by using a Matrix 9600 direct beta counter (Packard, Meriden, CT; cpm range from 10 to 35,000).

### Detection of Antibody-secreting Cells by ELISpot

Ninety-six-well multiscreen plates (mixed cellulose esters membrane) (Millipore, Billerica, MA) were coated overnight at 4°C with anti-mouse IgG (H+L) Ab (2 µg/mL) (Jackson ImmunoResearch, WestGrove, PA) or with histones (3 µg/ml) diluted in PBS. Histones were prepared from calf thymus and purified as described previously [54]. There is no change in the primary structure of calf and mouse core histones. The content of histone fraction was checked by 18% SDS-polyacrylamide gel electrophoresis. After washings with PBS, the ELISpot plate membrane was saturated for 1 h with complete medium. Serial dilutions of spleen mononuclear cell suspensions or FACS isolated cell subsets were then incubated for 6 h at 37°C in a CO_2_ incubator. Plates were then washed with PBS and PBS containing 0.05% (v/v) Tween (PBS-T) before incubation with anti-mouse IgG (Fcγ-specific; Jackson ImmunoResearch) or anti-mouse IgM (Fcμ-specific; Jackson ImmunoResearch) or anti-mouse IgA (Fcα-specific; SouthernBiotech, Birmingham, Alabama, USA) secondary Ab, each diluted 1/10,000 for 12 h at 4°C, and subsequently with alkaline phosphatase-conjugated extravidin (Sigma) for 1 h at 37°C. Final detection was carried out by addition of 5-bromo-4-chloro-indolyl phosphate and nitro-blue tetrazolium chloride substrate (BCIP-NBT; Sigma) and reaction was stopped with water when spots were clearly visible. Spots were counted with a Bioreader® 4000 (BioSysGmbH, Karben, Germany). Results were expressed as the number of Ab-secreting cells/million of cells and were calculated as follows: (number of spots/well – background measured in control uncoated wells) x dilution factor of the cell suspension.

### Chemotaxis Assay

CD138^+^ MACS-enriched cells (5×10^5^) were introduced in the upper chamber of 24-well plates containing Transwell inserts (6.5 mm diameter, 5 µm pore size; Corning, Acton, MA). The lower Transwell chamber was filled with 600 µL complete medium supplemented with either CXCL9, CXCL10, CXCL11 or CXCL12 chemokine (100 nM; R&D Systems). After a 2 h-incubation, cells that had migrated in the lower chamber were collected and labeled with anti-CXCR3 and anti-CD138 Ab (or alternatively anti-CD138 and anti-IA^d^ Ab) for flow cytometry analysis. The same procedure was followed for competition experiments, except that the chemokine used as a competitor was added together with cells in the upper chamber (100 mM).

### Quantitative Real-time PCR

Total RNA was prepared from isolated spleen cell subsets or from frozen-crushed kidney powder using Tri Reagent (Sigma-Aldrich) and purified using RNeasy® Mini Kit (Qiagen, Germantown, MD) according to the manufacturer’s instructions. cDNA was synthetized by extension of a mix of oligo(dT) and random primers with ImProm-II™ reverse transcriptase (Promega, Fitchburg, WI). The relative quantity of each transcript was normalized according to the mean of the expression of three different housekeeping genes, namely glyceraldehyde 3-phosphate dehydrogenase (GAPDH), hypoxanthine guanine phosphoribosyl transferase (HPRT), and β-actin. Primer sequences (forward/reverse) for *prdm1*, *xbp1*, *pax5 and ifnγ*, were designed with the Primers3 software (http://packages.debian.org/fr/sid/primer3) and are available upon request. All amplification reactions were performed in a total volume of 25 µl using either a Thermocycler sequence detector (Mx4000 or Mx3005P, Stratagene, La Jolla, CA) or a StepOnePlus ™ (Applied Biosystems, Carlsbad, California) with Mesa Green qPCR MasterMix Plus for SYBR Assay Low ROX (Qiagen or Eurogentec, Seraing, Belgium). Data were analyzed using the software tool REST (http://www.gene-quantification.de/download.html). The “Mouse Chemokines & Receptors” RT^2^ Profiler™ PCR Array as well as CXCL9, CXCL10 and CXCL11 primer sequences (Qiagen, accession numbers NM_008599.4, NM_021274.1 and NM_019494.1, respectively) and CXCR3 and CXCR4 primer sequences (SaBiosciences, Frederick, MD) were used according to the manufacturers’ instructions.

### Electron Microscopy

FACS-sorted cell subsets were fixed using 2.5% glutaraldehyde for 1 h. They were rinsed, post-fixed using 0.5% osmium tetroxide for 1 h, dehydrated in ascending series of dilution of ethanol, impregnated in grading concentrations of Epon (LX112 kit, Ladd, Williston, VT) in propylene oxide, incubated overnight and finally embedded in Epon after curing at 60°C for 48 h. In some experiments, mice were perfused with a mixture of formaldehyde (4%) and glutaraldehyde (0.2%). Kidneys were taken out and cut using a vibrating microtome. Sections (50 µm) were processed for pre-embedding labeling using unlabeled anti-CXCR3 Ab (clone 220803, R&D) followed by goat anti-rat IgG conjugated to ultra small gold particles (Aurion, Wageningen, The Netherlands). Sections were then post-fixed for 10 min in 0.5% osmium tetroxide and dehydration and embedding were processed as described previously [55]. Ultrathin sections (70 nm) were stained with uranyl acetate 4% for 15 min, with lead citrate for 2 min and examined by transmission electron microscopy (Hitachi H600). Images were acquired using a CCD camera (Hamamatsu, Hamamatsu City, Japan).

### Immunofluorescence

Kidney specimens were fixed with formaldehyde 4% and embedded in paraffin. Sections (4 µm) were deparaffinized, microwaved in 10 mM EDTA (pH 8) for 30 min, incubated in 4% (v/v) normal goat serum and overnight with appropriate dilutions of primary Ab and control sera. Kidney sections were stained with a mixture of the following Ab: monoclonal rat anti-CXCR3 Ab (clone 220803, R&D), polyclonal rabbit anti-CD138 Ab (Invitrogen, Cergy-Pontoise, France) and polyclonal goat anti-mouse IgG-FITC Ab (SBA). After washing, sections were incubated for 2 h with a mixture of the following secondary Ab: biotinylated goat anti-rat IgG (H+L) Ab (Jackson Immunoresearch) and polyclonal goat anti-rabbit Alexa ^647^ Ab (Molecular Probes, Invitrogen) followed, after washing, by a 30-min incubation in streptavidin Alexa^546^ (Molecular Probes). Sections were mounted with fluorescent Mounting Medium (Dako, Glostrup, Denmark) and observed with an inverted fluorescent microscope (Axiovert 200M, Zeiss) equipped with a CCD camera.

### Statistics

Statistical analyses were performed using Student t test and Mann–Whitney U test. For all tests, p<0.05 was considered statistically significant.

## Supporting Information

Figure S1 **Both CXCR3^+^CD3^+^ and CXCR3^+^CD3^−^ cells are located in inflamed kidneys.** CXCR3-expressing CD3^+^ cells (bottom images) and CXCR3-expressing CD3**^−^** cells (upper images; potentially CD138^+^ cells) were detected by immunofluorescence on paraffin-embedded kidney sections from proteinuria-positive NZB/W mice (bottom images). Sections were labelled with CXCR3-specific (red, clone 220803, R&D) and CD3-specific (green, polyclonal rabbit-anti-CD3, DAKO) Ab. Double staining as well as nucleus DAPI staining (blue) are shown in the overlay pictures. Scale bars: 50 µm (low magnifications) or 10 µm (enlarged images).
(TIF)
