# Supplementary figures and images for: Early Differentiated CD138highMHCII+IgG+ Plasma Cells Express CXCR3 and Localize into Inflamed Kidneys of Lupus Mice
Source: PLoS One. 2013 Mar 8;8(3):e58140. doi: 10.1371/journal.pone.0058140 (PMC3592892; doi:10.1371/journal.pone.0058140)

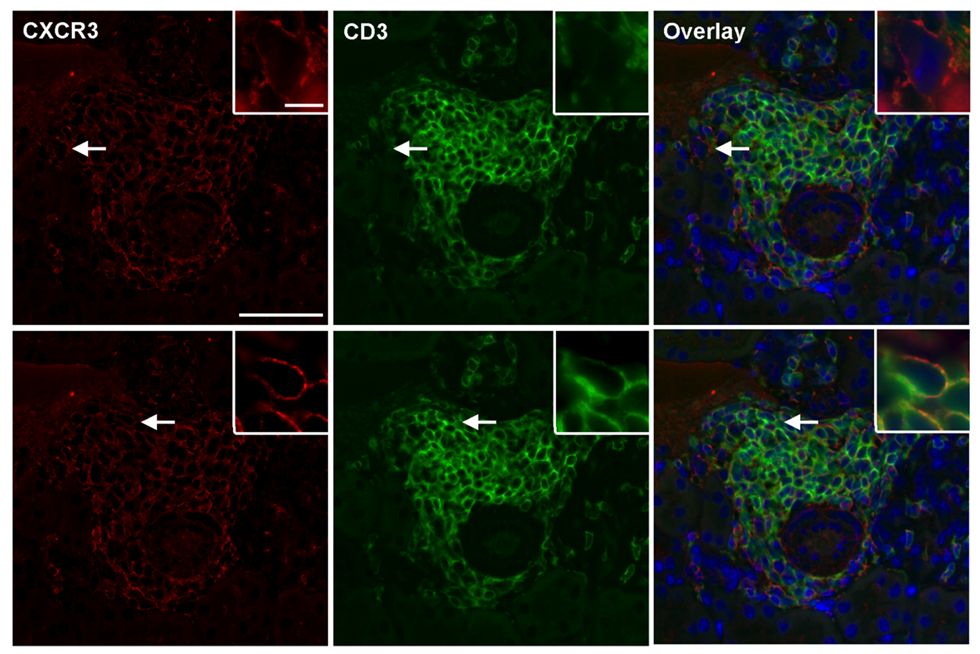

Supplement: Figure S1 — Both CXCR3+CD3+ and CXCR3+CD3− cells are located in inflamed kidneys. CXCR3-expressing CD3+ cells (bottom images) and CXCR3-expressing CD3− cells (upper images; potentially CD138+ cells) were detected by immunofluorescence on paraffin-embedded kidney sections from proteinuria-positive NZB/W mice (bottom images). Sections were labelled with CXCR3-specific (red, clone 220803, R&D) and CD3-specific (green, polyclonal rabbit-anti-CD3, DAKO) Ab. Double staining as well as nucleus DAPI staining (blue) are shown in the overlay pictures. Scale bars: 50 µm (low magnifications) or 10 µm (enlarged images). (TIF) [file pone.0058140.s001.tif]
